# Natural Antioxidant Potential of Melon Peels for Fortified Foods

**DOI:** 10.3390/foods12132523

**Published:** 2023-06-28

**Authors:** Filomena Monica Vella, Roberto Calandrelli, Domenico Cautela, Bruna Laratta

**Affiliations:** 1 National Research Council (CNR), Institute of Biosciences and BioResources (IBBR), Via P. Castellino, 80131 Naples, Italy; filomenamonica.vella@cnr.it; 2 National Research Council (CNR), Institute of Research on Terrestrial Ecosystems (IRET), Via P. Castellino, 80131 Naples, Italy; roberto.calandrelli@cnr.it; 3 Department of Theoretical and Applied Sciences, e-Campus University, 22060 Novedrate, Como, Italy; domenico.cautela@uniecampus.it

**Keywords:** *Cucumis melo*, peels, polyphenols, antioxidant activity, fortified foods, by-product exploitation

## Abstract

Agricultural and food waste recycling reduces natural resource losses, contributing significantly to the development of new green markets through the creation of redesigned products. In order to cycle valuable molecules, the peels from Italian cantaloupe (*Cucumis melo* L.) cultivars were studied and successfully characterized for high-added biomolecules to verify their possible exploitation as wealthy biomasses. Peels were investigated for their cell wall-modifying and browning enzymes, as well as for total polyphenols, *ortho*-diphenols, flavonoids, tannins, and antioxidant properties. The results of the analyses displayed great promise in one of the three cultivars investigated. Later on, a preliminary study using the best peel extract as a dietary supplement was carried out by preparing fortified seawater to enhance its antioxidant power. The effects of storage time (60 days) were examined at two temperatures through the determination of the stability of the polyphenol content. The kinetic parameters of degradation were also calculated. The “enriched sea water” retained great antioxidant activity in refrigerated conditions, demonstrating that there is good potential for melon by-products to add their natural compounds for food fortification. These findings may provide valuable data for scale-up, from the lab to the pilot or industrial application.

## 1. Introduction

The valorization of organic waste has attained great consideration in the last few decades due to the increase in sustainable policy regulations driven by the growing environmental awareness concerning the disposal of organic residues. Waste food valorization is a valuable way to transform all remaining materials (steams, leaves, flowers, seeds, and peels) into new valuable products. Minimizing food waste through the recycling of by-products is one of the choices that has recently focused European policies. Different management methods are available to avoid environmental problems while at the same time helping the economy and society, including the use of food waste for making new food products [1,2,3]. Reutilization through a bio-refinery concept represents a solution to the reduction of food waste’s volume and absolutely comes with the objectives of a circular economy based on the paradigm of reduction, reuse, and recycling [4,5,6,7]. Thus, information on the compositional value of waste parts is a crucial and primary aspect of the recovery program based on circular action.

Melons of the Cucurbitaceae family are economically important crops widespread throughout the world. The melons are cultivated in countries with temperate climates, from America and Europe to arid regions in Asia and Africa [8]. Melon’s annual production has been around 30 million tons for the last ten years, with China as the leading producer, followed by Turkey, Iran, and India [8,9]. Melons are generally consumed fresh-cut, in salads and drinks, cooked or processed in soup, jam, dessert, and dried. Therefore, a large quantity of inedible parts such as peel and seeds are discarded during all phases of supply chain handling, including harvesting, transport, storage, marketing, and processing, reaching approximately 20 million tons of biomass discarded per year worldwide [3]. Especially the netted cantaloupes are in demand because they possess high levels of several interesting molecules for human life. Cantaloupe fruit (var. *reticulatus*) is not only rich in carotenoids, vitamin C, and microelements such as potassium and magnesium [5,6,10], but it is also a potential source of natural compounds that inhibit oxidation and therefore have potential use in food and nutraceutical applications [5,6,10,11]. Among antioxidants, polyphenols are a class of specialized metabolites, of which cantaloupe is bounteous. They play a crucial role in the physiological pathways of fruits, and both abiotic (i.e., UV radiation, heat, heavy metals) and biotic (i.e., herbivores, pathogens) stresses trigger their production as molecules to be involved in plant protection [12,13]. Thus, polyphenols represent a defensive mechanism to cope with multiple constraints, and their increasing biosynthesis is finalized to overcome these adverse situations [12,13].

The cantaloupe peels are usually unexploited; nevertheless, they represent a large part of the fruit weight (around 25–44%), retaining a high concentration of phytochemical compounds [5,14,15]. The hydromethanolic extract of peel from Mexican varieties of market melons was studied and showed a large quantity of polyphenols, particularly isovanillic, 3-hydroxybenzoic, chlorogenic, and neochlorogenic acids. All cultivars express high levels of luteolin-7-*o*-glucoside and small amounts of two other flavonoids, apigenin-7-α-galactoside and quercetin-3-galactoside [16]. Other studies, almost all focusing on fruit waste parts, have been carried out, but only Gómez-García characterized the bioactive compounds and activity of the peels of *inodorus* melons, and Ganji et al. reported certain results for the extraction of peel powders from Mexican cultivars [15,16]. Fundo et al. [10] evaluated the potential of melon peel in terms of relevant constituents, and Miller et al. investigated all parts of the *inodorous* melon group, questioning the total antioxidant activity and the bioactive compounds [14]. This latter indicates the wasted materials, peels and seeds, as potential sources of antioxidants (carotenoids, polyphenols, and chlorophylls), but research on the peels of cantaloupe melon is still weak and hence deserves to be continued.

Therefore, the first objective of this work was to evaluate the compositional and bioactive characteristics of peels from three commercially distributed cultivars in Italy in order to establish the effective reuse of these melon by-products. To get comprehensive information about peel composition characteristics and bioactive properties, the enzymatic activities related to antioxidant and maturation processes were also investigated. To the best of our knowledge, studies about oxidative and degradative activities have never been conducted on cantaloupe peels [5,17], despite several patented active supplements from melon pulp and juice that have been developed recently. SOD B Dimpless^®^ and Extramel^®^ are nutraceutical products based on natural extracts of melons, to mention some of them currently on sale, and containing active superoxide dismutase that claim a list of health benefits [18,19].

Only recently, Vella et al. reported differences in enzyme activities in three melon varieties during ripening that were dependent on cultivars and maturity stages [17]. Hence, the plant antioxidant system, comprising enzymes such as the polyphenol oxidase (PPO, EC 1.14.18.1) and the peroxidase (POD, EC 1.11.1.7), which are enzyme indicators of the quality weakening of fruits, is considered. Moreover, superoxide dismutase (SOD, EC 1.15.1.1) and catalase (CAT, EC 1.11.1.6), as antioxidant group enzymes, are analyzed together with the hydrolytic enzymes pectin methylesterase (PME, EC 3.1.1.11) and polygalacturonase (PG, EC 3.2.1.15).

The growing request for recycling and/or transformation of vegetal materials into new valuable foods has led to the exploitation of such waste extracts for their application as nutritional supplements in food while, at the same time, reducing organic waste volume. Recently, Gómez-García et al. demonstrated the promising prebiotic effect of peel extract for the future development of functional ingredients from melon by-products [15]. To transform melon materials into a potential edible matrix with health benefits, these parts were treated with a freeze-drying process [20].

Nonetheless, cantaloupe peel extracts have not been extensively considered for their potential application as functional additives with beneficial properties, thereby mitigating the harmful effects of melon waste. Therefore, owing to the richness in phytochemicals, in this study, a new formulation of seawater used in cooking was also specifically tested with the addition of the best peel extract. Seawater was chosen as the model because many countries around the world use it to prepare their typical foods. For example, the Japanese use seawater to coagulate tofu; in Greece, where it is still common practice in some parts of the country, olives are treated for their tannins by immersion in the sea. Many bakers rely on seawater as an essential ingredient in bread and related products. Cheesemakers in New Zealand use seawater to curdle their milk. Koreans used to preserve wilted vegetables in seawater, a precursor to kimchi.

In addition, it has been shown in the literature that the lower salt content of seawater may be used as a substitute for salt in low-sodium diets (i.e., for hypertension, kidney disease, etc.) thanks to its mineral content while also providing good taste [21]. Seawater consists mainly of water (96.5%) and minerals (2.5%), including magnesium, potassium, iodine, and small amounts of other safe substances. The supplementation of seawater with compounds from melon peels with nutraceutical activity could be a useful strategy to contribute to or help in cases of degenerative diseases caused by oxidative stresses, as well as to be taken into consideration by people with a controlled sodium diet, often associated with mineral deficiency.

## 2. Materials and Methods

### 2.1. Vegetal Materials

Cantaloupe fruits (*C. melo* L. var. *reticulatus*) were supplied by Enza Zaden Italia S.r.l. (Tarquinia—VT, S.S. Aurelia Km 96.71, Italia).

All experimental analyses were carried out on three extended shelf life (ESL) varieties (Eminenza, SV7881, and Iperione). The Eminenza variety is characterized by limited storage ability caused by rapid overripening and faster loss of firmness. Cultivar SV7881 has very good storage capacity, and the Iperione variety has an excellent shelf life with limited over-ripening and softening, as stated in the information provided by the above international company.

The three varieties were all gathered at the same commercial maturity, measured as °Brix (Eminenza 13.3, SV7881 13.7, and Iperione 14.5). Fruits with uniform size and the absence of visual blemishes and/or diseases were harvested and immediately transported to the laboratory. After harvesting, fruits were rinsed with water to remove dirt and dust. Peels from three different fruits per cultivar were manually removed with a knife, cut into small pieces (1 cm × 1 cm), mixed to prepare a statistically significant sample, and immediately frozen. For the biochemical analysis, frozen peels were further lyophilized for 24 h, powdered using a food mixer, and kept at −20 °C until extractions were performed [22]. Enzymatic activities were performed on freshly frozen peels.

### 2.2. Reagents and Standards

All solvents and reagents were of analytical grade: buffers, acids, bases, salts, dithiothreitol (DTT), bovine serum albumin (BSA), phenolic acid standards, L-ascorbic acid, catechol, guaiacol, enzyme standards, and galacturonic acid were all acquired from Sigma Aldrich S.r.l. (Milan, Italy).

### 2.3. Phytochemicals and Antioxidant Activity

The extraction of phytochemicals was carried out as described by Vella et al. [17,22]. Briefly, a fine powder quantity of peels, approximately 200 mg, was extracted by adding 10 mL of 70% ethanol (ratio 1:50 *w*/*v*) for 6 h in an ultrasonic bath at 50 °C. After centrifugation at 13,000× *g* for 10 min at 4 °C, the extracts were dried using a rotary evaporator. All extracts were analyzed in triplicate to evaluate total polyphenols, *ortho*-diphenols, flavonoids, and tannin content, as previously described by Vella et al. [17].

The Folin-Ciocalteu method was used to determine total polyphenols [23]. In brief, extracts (150 µL), Folin-Ciocalteu reagent (750 µL), and 600 µL of Na_2_CO_3_ at 7.5% (*w*/*v*) were mixed. After 1 h of incubation, the absorbance at 765 nm was determined. Polyphenol amounts were expressed as µg of gallic acid equivalents (GAE) per mg of extract (dry weight), using a calibration curve with gallic acid (R^2^ = 0.9909).

*Ortho*-diphenol content was determined using the Arnow test [24]. Shortly, 400 µL of extracts, with 400 µL of 0.5 M HCl, 400 µL of 1.45 M NaNO_2_–0.4 M Na_2_MoO_4_, and 400 µL of 1 M NaOH, were assembled in a spectrophotometric vial. The absorbance at 500 nm was read, and the *ortho*-diphenols were expressed as µg of caffeic acid equivalents (CAE) per mg of extract (dry weight) through a calibration curve with caffeic acid as standard (R^2^ = 0.9921).

Flavonoids were determined according to the colorimetric test based on aluminum complex formation [25]. In brief, different amounts of extracts were combined with 1.25 mL of distilled water and 75 µL of NaNO_2_. After incubation for 5 min, 150 μL of AlCl_3 ×_ 6H_2_O were added, and the reaction was ended by 1 M NaOH. The absorbance at 510 nm was recorded, and results were expressed as µg of catechin equivalents (CE) per mg of extract (dry weight), using (+)-catechin as standard in the calibrating curve (R^2^ = 0.9940).

Tannins were evaluated according to Vella et al. [22]. The extracts were kept in a bath at 30 °C for 1 h with bovine serum albumin (BSA). The supernatant, the fraction without tannins, was centrifuged at 13,000 *g* for 10 min at 4 °C and then collected to be analyzed using the Folin-Ciocalteu assay. Consequently, tannins were determined by subtracting from the amounts of polyphenols determined before BSA precipitation the amounts after protein downfall. Tannins were expressed as µg of gallic acid equivalents (GAE) per mg of extract (dry weight) (R^2^ = 0.9909).

The antioxidant properties in extracts were evaluated through two assays: the Ferric Reducing Antioxidant Power (FRAP) and the DPPH (2,2-diphenyl-1-picrylhydrazyl) radical-scavenging activity. All samples were in triplicate. The FRAP reagent was mixed with extracts, and the absorbance was read at 593 nm, as described by Benzie and Strain [26]. The antioxidant power was calculated from the calibration curve with L-ascorbic acid, and results were indicated as µg of ascorbic acid equivalents (AAE) per mg of extract (dry weight) (R^2^ = 0.9918).

The DPPH assay, according to the procedure of Blois, allowed for the determination of the radical scavenging activity (RSA) of the extracts [27]. Therefore, different amounts of extract were mixed with the DPPH methanolic solution. The decrease in absorbance at 517 nm in solution was determined continuously. The %RSA was calculated as DPPH discoloration by means of the equation:

$$\%RSA=\left[\frac{\left(A_{DPPH}-A_{s}\right)}{A_{DPPH}}\right]\times 100$$

where A_S_ is the absorbance of the solution when the extract was added, and A_DPPH_ is the absorbance of the DPPH solution. The EC_50_ value was obtained from the graph of %RSA vs. the extract concentrations in mg/mL.

### 2.4. Enzymatic Activities

The enzymatic extracts of PPO, POD, SOD, CAT, PME, and PG were obtained according to Vella et al. [17]. Briefly, 7 g of frozen peels were cut and homogenized in phosphate buffer 0.1 M (pH 7.5) and 1 mM dithiothreitol (DTT). The PME and PG activities were extracted with the same buffer but added to 1 M NaCl. The mixture was stirred at 4 °C for 1 h and then centrifuged at 4000× *g* for 20 min at 4 °C. Supernatants were filtered, and the clarified solutions obtained were used as a crude extract for enzymatic determination. The enzyme activities were expressed in units (U), defined as the amount of enzyme required to produce 1 µmole of product per minute. The protein content was determined on extract by the method of Bradford [28], with BSA as the standard at 595 nm. The specific activity was reported in U per µg of protein (R^2^ = 0.9930).

The method used to determine PPO has been performed in phosphate buffer 100 mM, pH 6.8, and 0.1 M catechol (just prepared). The assay mixture was incubated for 3 min at room temperature (RT), and crude extract (50 µL) was added. Moreover, the reaction was monitored at 412 nm for 2 min at RT [17].

POD activity was determined on a mixture composed of phosphate buffer 100 mM pH 6.8, 3% hydrogen peroxide, and 4% guaiacol, freshly assembled. After 3 min of incubation at RT, 50 µL of crude extract was added. The reaction was examined at 470 nm for 2 min at RT [17].

The determination of CAT activity using 200 µL of crude extract has been achieved spectrophotometrically by the addition of 0.036% hydrogen peroxide in 50 mM phosphate buffer at pH 7.0. The absorbance decrease at 240 nm was followed for 2 min at RT [17].

Activity determination of SOD was performed at 450 nm by using a tetrazolium salt to detect superoxide radicals (SOD activity assay kit, Biovision—California, CA, USA). One unit of SOD is the amount of enzyme that catalyzes 50% dismutation of superoxide radicals [17,29].

The activity of PME was measured according to the Hagerman and Austin method with a slight modification [30,31]. Briefly, an aliquot of crude extract (from 1 to 10 µL) was added to 1 mL of 1% pectin solution (from citrus peels; galacturonic acid > 74%, methoxy groups > 6.7%) with 0.1 g/L solutions of bromothymol blue as the indicator. The reaction started by adding the crude extract, and the absorbance rate decreased to 620 nm. PME activity of 1 U corresponded to an absorbance decrease of 0.1 per minute.

The PG assay is based on the release of reducing groups by the enzyme action on pectin at a low degree of esterification. In this work, it was measured spectrophotometrically using the Miller method with the dinitrosalicylic (DNS) acid reagent [32]. Briefly, crude extract was incubated with a 0.5% poly-galacturonic acid solution (MW 25,000–50,000) in phosphate buffer 50 mM, pH 7.0. After 2 min of incubation at RT, the same volume of the DNS reagent was added to the test tube, and the mixture was boiled for 5 min. The reaction ended in ice, and the absorbance was recorded at 546 nm [32].

### 2.5. Seawater Fortification

Sea water for food use, named “Marentìa di Sardegna”, was produced by an Italian company (Abba Blu S.r.l., San Sperate—CA, Italy), and supplied by Sapori Blu S.r.l.s. (Portici—NA, Italy). Characterization for salinity and chemical composition, as well as microbiological parameters, were reported on the company data sheet.

Functional seawater was produced by adding a powder extract of peels, 5 mg/mL, from the Iperione cultivar, as explained in Section 2.3. The stability of functionalized water was studied at two temperatures, RT and 4 °C, through the study of the stability of phenolic compounds. Measurements of total polyphenol contents were performed in triplicate at 0, 1, 3, 7, 10, 20, 30, 50 and, 60 days.

The stability of fortified seawater gave first-order degradation kinetics for phenolics following a model described by Costa et al. [33].

The reaction was expressed by the following Equation (1):

ln (C_t_/C_0_) = −kt (1)

where C_t_ is the phenolic content at t days of storage, C_0_ is the initial phenolic content, k is the reaction rate constant, and t is the number of days of storage.

The half-life time (t_1/2_) of a first-order reaction can be calculated using Equation (2).

t_1/2_ = ln (2)/k (2)

### 2.6. Statistical Analysis

All results were expressed as mean ± standard deviation (SD) on samples that were analyzed in triplicate. Means, SD, calibration curves, and linear regression analyses (R^2^) were determined using Microsoft Excel 2013 (Microsoft Corporation, Redmond, WA, USA).

## 3. Results

This section may be divided by subheadings. It should provide a concise and precise description of the experimental results, their interpretation, and the experimental conclusions that can be drawn.

### 3.1. Bioactive Compounds and Antioxidant Activity

To provide useful information on the potential reuse of peels from Italian cantaloupe cultivars, this work was carried out in preparation for an in-depth understanding of their bioactive compounds and enzymatic activities (related to softening and browning processes). Melon fruits have been investigated for phytochemicals because, beyond their physiological role in plants with protective purposes against predators, they exert antioxidant and biological properties that can be used as food supplements.

The peels of three varieties were eco-friendly extracted with hydroalcoholic solutions and then characterized for their principal biologically active compounds, total polyphenols, *ortho*-diphenols, flavonoids, and tannins, as well as antioxidant activities. All experiments were always carried out on fruits harvested at the commercial maturity stage, and the results are reported in Table 1.

The present results have contributed to increasing our understanding of the secondary metabolism of cantaloupe peels, demonstrating that there are significant quantitative differences (*p*-value < 0.05) in these compounds among the cultivars studied.

In Iperione’s peels, the highest total polyphenol content was demonstrated at commercial maturity, with 10.35 ± 0.01 µg GAE/mg, followed by the cultivar SV7881 with 9.84 ± 0.01 µg GAE/mg, and finally the cultivar Eminenza with 6.70 ± 0.01 µg GAE/mg. These results are approximately three times higher than those accounted for by Ismail et al. [11]. In a previous work, Vella et al. [22] found a polyphenol content of 25.48 mg GAE/g in peel extracts of some unknown melon cultivars. Instead, cultivars Iperione, SV7881, and Eminenza contain fewer polyphenols compared to the former study. The very different values could be related to several factors, among them those regulating the growth of the fruit, the genotype, the degree of ripeness at harvesting, and the environmental and geospatial conditions [5,11,22,34]. Other studies pointed out melon rinds as rich sources of bioactive compounds with relevant antioxidant activity. Sroy et al. [20] found 2251.2 µg/g (on a dry basis) of total polyphenols, which is 5 times lower than the results we obtained with Iperione.

Among the polyphenols, *ortho*-diphenols are the most important for healthy purposes because they can enhance radical stability by forming an intramolecular hydrogen bond between hydrogen and phenoxyl radicals. *Ortho*-diphenol content in peel extracts of these cultivars followed the same trend shown for polyphenols. The highest contents were found for Iperione peels (3.38 ± 0.02 µg CAE/mg), followed by SV7881 (3.03 ± 0.03 µg CAE/mg), and lastly, Eminenza (1.93 ± 0.03 µg CAE/mg). Vella et al. [22] extracted the *ortho*-diphenols from *C. melo* peels for the first time, establishing a huge value of 17.86 CAE mg/g. Unlike this latter study, the divergences could be attributed to different cultivars used in this work [23].

Concerning flavonoids, the peels from the Iperione cultivar showed a maximum value of 7.02 ± 0.09 µg CE/mg. Lower values were obtained for peels from SV7881 and Eminenza of 3.64 ± 0.10 µg CE/mg and 3.25 ± 0.06 µg CE/mg, respectively (Table 1). For this class of phytochemicals, these results are better than those reported in the literature thus far [11]. However, flavonoid values are less than those found in our former work on cantaloupe peel recovery [22], similarly to the polyphenol contents. These differences could be attributed to the fact that flavonoids extent differs depending on fruit maturation, plant physiology state, and seasons [34].

The last bioactive molecules examined were tannins, which, together with total polyphenols, *ortho*-diphenols, and flavonoids, had the same behavior. Therefore, the top amount of tannins occurred in Iperione peels with 5.21 ± 0.02 µg GAE/mg; next followed the SV7881 with 3.81 ± 0.01 µg GAE/mg; and lastly, the Eminenza with 3.07 ± 0.04 µg GAE/mg (Table 1). This is the first phytochemical study on the melon extract reporting the presence of tannins, and they are consistent with some of our previous work [5,22].

The study of the antioxidant properties in the cantaloupe cultivars was carried out through the FRAP assay and the DPPH radical scavenging test, as reported in Table 1. Particularly, the peels of Iperione displayed the best antioxidant power of 4.90 ± 0.03 µg AAE/mg, measured by the FRAP assay, followed by SV7881 with 3.79 ± 0.01 µg AAE/mg and Eminenza with 2.45 ± 0.02 µg AAE/mg. Since both assays give overall information on the antioxidant power (FRAP) and quenching ability (DPPH) of an antioxidant molecule, according to the DPPH assay, the highest content of polyphenols for each cultivar corresponded to the lowest EC_50_. Hence, Eminenza peels show 14.41 ± 0.66 mg/mL, SV7881 10.57 ± 0.16 mg/mL, and lastly, Iperione 10.06 ± 0.13 mg/mL. These results are similar to data reported by Ismail et al. [11] that found an EC_50_ of 9.58 ± 0.37 mg/mL, indicating that the DPPH radical scavenging activity of cantaloupe extracts is highly related to the phenolic compounds present in the extracts.

Considering the overall data obtained, total polyphenols, *ortho*-diphenols, flavonoids, tannins, and antioxidant activity, the peels of one cultivar out of the three, Iperione, display the best qualities in terms of polyphenol contents and antioxidant activity. It is worth noting that polyphenols are molecules with well-recognized activity towards reactive oxygen species (ROS), produced in fruits during ripening. From this perspective, these first outcomes demonstrate that the peels of Iperione, possessing the best characteristics and biological properties, could be a potential source of useful and valuable phytochemicals. In this way, a non-edible part with no economic value, usually discarded in the environment, can be incorporated into different products (e.g., cakes, beverages, yogurts, etc.), elevating their nutritional profile and producing a new food product [33].

### 3.2. Enzymatic Activities

During the last decade, efforts to valorize food by-products have increased demands for testing different extraction techniques to isolate beneficial enzymes, for instance, from melon pulp and juice. In this context, it was demonstrated that the oral daily intake of melon juice rich in SOD helps to reduce symptoms of body stress and fatigue [18,19]. In the human body, defenses against free radicals are due to the synergic actions of the inner SOD and CAT antioxidant enzymes. Since their finding, many studies have been published, and recently, the efficacy of melon’s SOD supply in controlled clinical studies has proven how the SOD provides preventive and restorative effects from oxidative stress and gives inflammation relief in almost all tissues [18].

Thus, the exploitation of cantaloupe peels has been carried out to search for a new variety of Cantaloupe melon naturally rich in SOD [17,18,29]. The assessment of the enzymatic activity is important for the reuse of peels as the principal source of health compounds instead of exploiting pulps. Furthermore, browning enzymes (PPO, POD, CAT, and SOD) are strictly involved in the degradation of phenolic compounds, while softening enzymes (PME and PG) influence the postharvest life of products [35,36].

Therefore, PPO, POD, CAT, SOD, PME, and PG were assessed in the peels of Eminenza, SV7881, and Iperione at the commercial maturity stage. To the best of our knowledge, this is the first time that this study has been carried out on cantaloupe peels. Appearance and senescence of fruits were strictly linked to color changes, and, in particular, PPO and POD enzymes are involved in the browning phenomenon [36,37,38,39].

**Figure 1:**
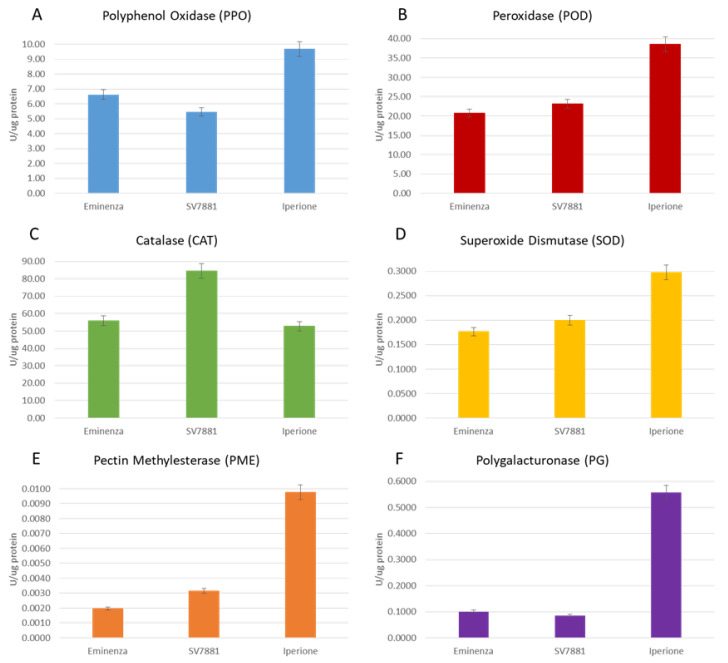
Enzymatic activities of Eminenza, SV7881, and Iperione melons: (**A**) polyphenol oxidase (PPO); (**B**) peroxidase (POD); (**C**) catalase (CAT); (**D**) superoxide dismutase (SOD); (**E**) pectin methylesterase (PME); (**F**) polygalacturonase (PG). Results are expressed as the mean ± standard deviation.

The PPO- and POD-specific activities of melon peels were reported at maturity stage in Figure 1A,B, respectively. Among the three cultivars, Iperione possesses the highest PPO-specific activity value (9.68 U/µg of protein). Eminenza follows with 6.62 U/µg of protein, and lastly, SV7881 has 5.46 U/µg of protein, a value lower by about 1.8-fold than Iperione. A high PPO activity like that registered in these cantaloupe cultivars can be related to an accelerated metabolic activity during a particular maturation phase, as reported by some authors [22,35]. In this respect, it is noteworthy that the wider the antioxidant system the cultivars have developed, the fewer browning effects can occur in the post-harvest period [36,37,38,39].

During ripening, respiration rate and ethylene production cause changes in metabolic pathways, such as chlorophyll degradation, softening, tissue senescence, and color variation, and in turn these cause the output of ROS, such as superoxide and hydroxyl radicals, as well as hydrogen peroxide. The latter compounds are substrates of detoxifying enzymes such as POD [39]. In this study, the maximum POD enzyme activity was 38.57 U/µg of protein in Iperione peels, a value that was about 1.6-fold greater than Eminenza (20.77 U/µg of protein) and 1.8-fold greater than SV7881 (Figure 1B).

CAT, one of the main enzymatic defenses against oxidative stress induced by senescence [40], was evaluated in cantaloupe peels, and the results are reported in Figure 1C. The specific activities recorded for CAT are 55.94, 84.48, and 52.69 U/µg of protein, respectively, for Eminenza, SV7881, and Iperione peels.

The activity of the SOD enzyme extracted from the peels of cantaloupe melons at five harvesting stages is depicted in Figure 1D. In Eminenza, the SOD specific activity is 0.1763 U/µg of protein; in SV7881, it is 0.1194 U/µg of protein; and in Iperione, it reaches the uppermost value of 0.2979 U/µg of protein. The Extramel^®^ patent [19] is for a *C. melo* protein extract with a SOD activity greater than 0.03 U/µg of proteins and a CAT activity greater than 0.045 U/µg of proteins. Within this perspective, results clearly show a significant improvement in the specific activities of oxidation enzymes here extracted from peels compared to the patented juice and pulp extraction [29].

The changes in the cell wall composition during ripening, resulting from the gradual solubilization of pectin polymers, drive the progressive loss of firmness, limiting fruit shelf-life [36,41]. In that regard, PME and PG are the main degrading enzymes involved, and therefore, their activities were explored in peels. In Figure 1E,F, the specific activities of PME and PG for Eminenza, SV7881, and Iperione cultivars, respectively, are reported. In particular, PME-specific activity was comparable between Eminenza (0.0020 U/µg of protein) and SV7881 (0.0031 U/µg of protein), while in Iperione this value is higher, reaching 0.0098 U/µg of protein. These results confirm the greater stability and longer shelf life of the Iperione cultivar. The latter feature is very important to preserve the quality of the market and the commercial choices of consumers. However, as indicated by research on recent literature data, PME activity may differ, decreasing or remaining constant, during the growth of fruits [30,35,36,40].

The PG enzyme, together with PME, helps to depolymerize pectin during the maturation process in fruits. The PG activity, obtained from peel extracts of Eminenza, SV7881, and Iperione, is shown in Figure 1F. The PG activity, obtained from peel extracts of Eminenza, SV7881, and Iperione, shows the same behavior described for PME. In fact, PG-specific activity in Eminenza was 0.1010 U/µg of protein, an amount comparable with that recorded in SV7881 of 0.0851 U/µg of protein. In Iperione, the PG activity was 0.5564 U/µg of protein, a value higher by 5.5 and 6.5 folds than Eminenza and SV7881 cultivars, respectively.

These findings suggest that this enzyme, together with PME, plays a key role in oxidative metabolism, polyphenol accumulation, cell wall integrity, and the regulation of cantaloupe varieties [10,34,36]. These results suggest that the Iperione cultivar, with excellent ripening characteristics and antioxidant properties, may have a positive impact on consumers and markets, leading to high commercial profitability. In fact, in the last decades, pectinases have been widely applied in various industries, such as the food, wine, textile, paper, and pulp industries, to mention a few [30,41,42].

### 3.3. Stability of Fortified Sea Water

Currently, health recommendations for lowering sodium intake in diets have prompted manufacturers to make many efforts to design new foods, like bread and other bakery products, by replacing NaCl with low-sodium sea salt [20]. These authors report that bread prepared with sea salt shows higher absorption features during baking due to the presence of different ions (MgCl_2_ and KCl) that impact protein solvation. These studies have encouraged the investigation of the promising peel extract as an ingredient for use in formulating reduced-sodium food products. In this work, seawater was fortified with the best crude peel extract obtained from cantaloupe to explore the likely valorization of this valuable by-product in health food markets [15,42]. The first part of our research demonstrated the availability of polyphenols and antioxidant enzymes contained in peel extracts, which are eco-friendly extracted and measured. However, to meet the needs of people who consume insufficient fruit and vegetables, the antioxidant complex of these extracts could be unstable and prone to loss of biological activity.

**Figure 2:**
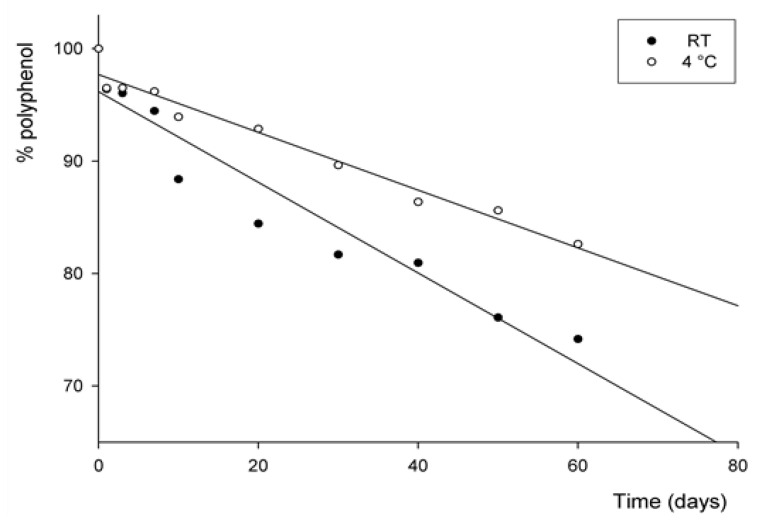
Stability of polyphenols after addition of peel extract to seawater during storage at two temperatures.

In this view, the stability of polyphenols was studied by comparing the total phenol contents at two temperatures, room temperature (RT) and cool temperature (4 °C), respectively. The experiments were carried out at two temperatures, storing the samples of fortified seawater for up to 60 days. Table 2 and Figure 2 show the results obtained, and, moreover, the parameters of the kinetic reactions are provided in Table 3.

Figure 2 presents the changes in total phenolic content of sea water added with peel extract during storage at two temperatures: room temperature (RT, 25 °C) and refrigerated (4 °C). Seawater at RT and at 4 °C followed a similar decay behavior for polyphenols. After 60 days of storage at RT, the polyphenol content in seawater was 43.82 µg/mL GAE, maintaining 74.18% of the initial content. Whereas at 4 °C, the amount present in seawater was 48.80 µg/mL GAE, retaining 82.62% of polyphenols after 60 days. Similarly, the trend at RT was the same after 30 days of storage, as reported in Table 2 and Figure 2. These results suggest a positive influence of low temperature on polyphenol stability during storage, as reported by Deng et al., who reported how polyphenol retention depends on different factors such as pH, temperature, light, oxygen, enzymes, and proteins, as well as interaction with other food constituents [14].

The degradation of polyphenols follows a first-order reaction that seems likely to be similar to that reported by Costa et al. [33]. Results indicate that the polyphenol degradation rate was linearly dependent on the concentration of polyphenols present in fortified seawater. As reported in Table 3, seawater at 4 °C presents the greatest half-life time of 217 days, while seawater at RT displays a half-life of 139 days.

According to the Food and Drug Administration (FDA), fortified foods have extra nutrients added by the manufacturer, but they are not automatically intended to replace nutrients that may have been lost during processing. The systems containing polyphenols underwent little degradation during storage at 4 °C, indicating that the polyphenols were stable under the conditions used. Therefore, since the stability of polyphenols is pronounced at 4 °C, it can be suggested that this enriched seawater be used for processed foods stored at cool temperatures, as natural degradation reactions do not occur or are slowed down under these conditions [14].

## 4. Conclusions

Bio-fortification of food products using eco-friendly extracts from food waste is often decisive in enhancing the overall quality of new products. Therefore, their analytical characterization in plant-based foods is crucial and a priority.

The aim of this study was to understand the recycling potential of cantaloupe peels as high-value-added compounds. To accomplish that goal, the phenolic composition, enzymatic activities, and antioxidant properties were initially evaluated in the peels of three cantaloupe varieties at commercial maturity. Therefore, one extract was employed as a source of phenolic compounds to be added to a new food formulation. Particularly fortified seawater was made, showing a significant increase in total phenolic content and even stability in antioxidant activity after storage at 4 °C for more than one month.

The study demonstrated that melon peel extract has the potential to be used as a functional food ingredient for aiding human health or prolonging the shelf life of foods.

## Figures and Tables

**Table 1:** Phytochemicals and antioxidant activity in Eminenza, SV7881, and Iperione.

Cultivar	Total Polyphenols (µg GAE/mg)	*Ortho*-Diphenols (µg CAE/mg)	Flavonoids (µg CE/mg)	Tannins (µg GAE/mg)	Antioxidant Power (µg AAE/mg)	EC_50_ (mg/mL)
Eminenza	6.70 ± 0.01	1.93 ± 0.03	3.25 ± 0.06	3.07 ± 0.04	2.45 ± 0.02	14.41 ± 0.66
SV7881	9.84 ± 0.01	3.03 ± 0.03	3.64 ± 0.10	3.81 ± 0.01	3.79 ± 0.01	10.57 ± 0.16
Iperione	10.35 ± 0.01	3.38 ± 0.02	7.02 ± 0.09	5.21 ± 0.02	4.90 ± 0.02	10.06 ± 0.13

Gallic acid equivalents = GAE, caffeic acid equivalents = CAE, catechin equivalents = CE, ascorbic acid equivalents = AAE. *p*-value < 0.05.

**Table 2:** Percent of total phenolic compounds (expressed as µg/mL GAE) present in seawater at RT and at 4 °C during 60 days of storage.

Day	µg/mL GAE—RT	µg/mL GAE—4 °C
0	59.07 ± 3.051	59.07 ± 2.1705
1	56.93 ± 0.513	57.07 ± 0.391
3	56.73 ± 0.265	57.01 ± 0.464
7	55.80 ± 1.163	56.82 ± 0.7335
10	52.21 ± 0.8675	55.49 ± 0.755
20	49.88 ± 0.0085	54.86 ± 0.8315
30	48.25 ± 0.6165	52.95 ± 0.154
40	47.82 ± 0.5685	51.02 ± 0.1995
50	44.94 ± 0.2775	50.57 ± 0.373
60	43.82 ± 0.269	48.80 ± 0.382

RT = 25 °C.

**Table 3:** Kinetic parameters for phenolic stability in seawater.

Formulation	Rate Constant (k)	t_1/2_ (Days)
Seawater + peel extract (RT)	0.0050	139.3
Seawater + peel extract (4 °C)	0.0032	217.8

RT = 25 °C.

## Data Availability

The datasets generated for this study are available on request to the corresponding author.

## References

[1] Sagar N.A., Pareek S., Sharma S., Yahia E.M., Lobo M.G. (2018). Fruit and vegetable waste: Bioactive compounds, their extraction, and possible utilization. Compr. Rev. Food Sci. Food Saf..

[2] Ben-Othman S., Jõudu I., Bhat R. (2020). Bioactives from agri-food wastes: Present insights and future challenges. Molecules.

[3] Rolim P.M., Seabra L.M.A.J., de Macedo G.R. (2020). Melon by-products: Biopotential in human health and food processing. Food Rev. Int..

[4] Rico X., Gullón B., Alonso J.L., Yáñez R. (2020). Recovery of high value-added compounds from pineapple, melon, watermelon and pumpkin processing by-products: An overview. Food Res. Int..

[5] Laratta B., Pignone D., Vella F.M., Ramadan M.F., Farag M.A. (2022). Leveraging the *Cucumis melo* wastes. Mediterranean Fruits Bio-Wastes—Chemistry, Functionality and Technological Applications.

[6] Silva M.A., Albuquerque T.G., Alves R.C., Oliveira M.B.P., Costa H.S. (2020). Melon (*Cucumis melo* L.) by-products: Potential food ingredients for novel functional foods?. Trends Food Sci. Technol..

[7] Galanakis C.M. (2012). Recovery of high added-value components from food wastes: Conventional, emerging technologies and commercialized applications. Trends Food Sci. Technol..

[8] International plant genetic resources Institute (2003). Descriptors for Melon (Cucumis melo L.).

[9] Faostat, Food and Agriculture Organization of the United States. http://www.fao.org/home/en/.

[10] Fundo J.F., Miller F.A., Garcia E., Santos J.R., Silva C.L.M., Brandão T.R.S. (2017). Physicochemical characteristics, bioactive compounds and antioxidant activity in juice, pulp, peel and seeds of cantaloupe melon. J. Food Meas. Charact..

[11] Ismail H.I., Chan K.W., Mariod A.A., Ismail M. (2010). Phenolic content and antioxidant activity of cantaloupe (*Cucumis melo*) methanolic extracts. Food Chem..

[12] Šamec D., Karalija E., Šola I., Vujčić Bok V., Salopek-Sondi B. (2021). The role of polyphenols in abiotic stress response: The influence of molecular structure. Plants.

[13] Sharma A., Shahzad B., Rehman A., Bhardwaj R., Landi M., Zheng B. (2019). Response of phenylpropanoid pathway and the role of polyphenols in plants under abiotic stress. Molecules.

[14] Miller F.A., Fundo J.F., Garcia E., Santos J.R., Silva C.L.M., Brandão T.R.S. (2020). Physicochemical and Bioactive Characterisation of Edible and Waste Parts of “Piel de Sapo” Melon. Horticulturae.

[15] Gómez-García R., Campos D.A., Oliveira A., Aguilar C.N., Madureira A.R., Pintado M. (2021). A chemical valorisation of melon peels towards functional food ingredients: Bioactives profile and antioxidant properties. Food Chem..

[16] Ganji S.M., Singh H., Friedman M. (2019). Phenolic content and antioxidant activity of extracts of 12 melon (*Cucumis melo*) peel powders prepared from commercial melons. J. Food Sci..

[17] Vella F.M., Calandrelli R., Laratta B. (2021). Influence of ripening on polyphenolic content, degradative, and browning enzymes in cantaloupe varieties (*C. melo* L.). Horticulturae.

[18] Saby M., Gauthier A., Barial S., Egoumenides L., Jover B. (2020). Supplementation with a Bioactive Melon Concentrate in Humans and Animals: Prevention of Oxidative Damages and Fatigue in the Context of a Moderate or Eccentric Physical Activity. Int. J. Environ. Res. Public Health.

[19] Ginoux J.P., Dreyer A., Roch P., Baccou J.C., Lacan D. (1997). Cucumis melo Protein Extract with Antioxidant Activity and Process for Preparing It, Cosmetic or Pharmaceutical Composition or Food Composition Containing Such an Extract. U.S. Patent.

[20] Sroy S., Miller F.A., Fundo J.F., Silva C.L., Brandão T.R. (2022). Freeze-Drying Processes Applied to Melon Peel: Assessment of Physicochemical Attributes and Intrinsic Microflora Survival during Storage. Foods.

[21] Simsek S., Martinez M.O. (2016). Quality of dough and bread prepared with sea salt or sodium chloride. J. Food Process Eng..

[22] Vella F.M., Cautela D., Laratta B. (2019). Characterization of polyphenolic compounds in cantaloupe melon by-products. Foods.

[23] Singleton V.L., Rossi J.A. (1965). Colorimetry of total phenolics with phosphomolybdic-phosphotungstic acid reagents. Am. J. Enol. Vitic..

[24] Arnow L.E. (1937). Colorimetric determination of the components of 3,4-dihydroxyphenylalaninetyrosine mixtures. J. Biol. Chem..

[25] Zhishen J., Mengcheng T., Jianming W. (1999). The determination of flavonoids contents in mulberry and their scavenging effects on superoxide radicals. Food Chem..

[26] Benzie I.F., Strain J.J. (1996). The ferric reducing ability of plasma (FRAP) as a measure of “antioxidant power”: The FRAP assay. Anal. Biochem..

[27] Blois M.S. (1958). Antioxidant determination by the use of a stable free radical. Nature.

[28] Bradford M.M. (1976). A rapid and sensitive method for the quantitation of microgram quantities of protein utilizing the principle of protein-dye binding. Anal. Biochem..

[29] Palma J.M., Pastori G.M., Bueno P., Distefano S., Del Río L.A. (1997). Purification and properties of cytosolic copper, zinc superoxide dismutase from watermelon (*Citrullus vulgaris* Schrad.) cotyledons. Free. Radic. Res..

[30] Laratta B., De Masi L., Minasi P., Giovane A. (2008). Pectin methylesterase in *Citrus bergamia* R.: Purification, biochemical characterization and sequence of the exon related to the enzyme active site. Food Chem..

[31] Hagerman A.E., Austin P.J. (1986). Continuous spectrophotometric assay for plant pectin methyl esterase. J. Agric. Food Chem..

[32] Miller G.L. (1959). Use of dinitrosalicylic acid reagent for determination of reducing sugar. Anal. Chem..

[33] Costa J.R., Monteiro M.J., Tonon R.V., Cabral L.M., Pastrana L., Pintado M.E. (2021). Fortification of coconut water with microencapsulated grape pomace extract towards a novel electrolyte beverage: Biological, sensorial and quality aspects. Future Foods.

[34] Menon S.V., Ramana Rao T.V. (2012). Nutritional quality of muskmelon fruit as revealed by its biochemical properties during different rates of ripening. Int. Food Res. J..

[35] Chisari M., Silveira A.C., Barbagallo R.N., Spagna G., Artés F. (2009). Ripening stage influenced the expression of polyphenol oxidase, peroxidase, pectin methylesterase and polygalacturonase in two melon cultivars. Int. J. Food Sci. Technol..

[36] Prasanna V., Prabha T.N., Tharanathan R.N. (2007). Fruit ripening phenomena—An overview. Crit. Rev. Food Sci. Nutr..

[37] Toivonen P.M., Brummell D.A. (2008). Biochemical bases of appearance and texture changes in fresh-cut fruit and vegetables. Postharvest Biol. Technol..

[38] Singh B., Suri K., Shevkani K., Kaur A., Kaur A., Singh N. (2018). Enzymatic browning of fruit and vegetables: A review. Enzymes in Food Technology.

[39] Moon K.M., Kwon E.B., Lee B., Kim C.Y. (2020). Recent trends in controlling the enzymatic browning of fruit and vegetable products. Molecules.

[40] Zimmermann P., Heinlein C., Orendi G., Zentgraf U. (2006). Senescence specific regulation of catalase in *Arabidopsis thaliana* (L.) Heynh. Plant Cell Environ..

[41] Farcuh M., Copes B., Le-Navenec G., Marroquin J., Jaunet T., Chi-Ham C., Van Deynze A. (2020). Texture diversity in melon (*Cucumis melo* L.): Sensory and physical assessments. Postharvest Biol. Technol..

[42] Gómez-Garcia R., Campos D.A., Aguilar C.N., Madureira A.R., Pintado M. (2020). Valorization of melon fruit (*Cucumis melo* L.) by-products: Phytochemical and Biofunctional properties with Emphasis on Recent Trends and Advances. Trends Food Sci. Technol..

